# Treatment expectations and depressive symptoms in an internet-based intervention for depression. A secondary analysis

**DOI:** 10.1016/j.invent.2025.100869

**Published:** 2025-08-28

**Authors:** Gwendolyn Wälchli, Laura Luisa Bielinski, Oliver Thomas Bur, Tobias Krieger, Jan Philipp Klein, Thomas Berger

**Affiliations:** a Department of Clinical Psychology and Psychotherapy, University of Bern, Switzerland; b Department of Psychiatry and Psychotherapy, Luebeck University, Luebeck, Germany

**Keywords:** Expectations, Early process predictors, Internet-based self-help, Depressive symptoms

## Abstract

**Background:**

Treatment expectations are known to influence therapy outcomes, but their role in internet-based interventions (IBIs) for depression remains unclear. While previous research has primarily focused on expectations as a *pre-treatment predictor* (PTP), emerging evidence suggests that *early process predictors* (EPPs), including evolving expectations during treatment, may provide more relevant insights into therapeutic outcomes.

**Objective:**

This secondary analysis of a factorial trial (Bur et al., 2022) investigates the role of treatment expectations as both a *pre-treatment predictor* and *early process predictor* in an internet-based intervention for mild to moderate depression. It also explores the temporal relationship between expectations and depressive symptoms, assessing whether earlier expectations predict later symptom severity and whether depressive symptoms influence subsequent expectations.

**Methods:**

Treatment expectancy was measured using the Credibility and Expectancy Questionnaire (CEQ-8; Devilly & Borkovec, 2000; German version: Walach et al. 2008) at baseline (T0), two weeks (T1), and four weeks (T2), while depressive symptoms were assessed using the Patient Health Questionnaire-9 (PHQ-9; Kroenke et al., 2001) at the same time points as well as post-treatment (T3). To analyze the relationship between treatment expectations (CEQ-8) and depressive symptoms (PHQ-9 post-treatment), simple regression models were conducted while controlling for baseline PHQ-9 scores. Multiple regression analyses were then used to examine whether CEQ-8 predicted PHQ-9 or vice versa. In addition, as a sensitivity analysis, a cross-lagged panel model (CLPM) was estimated to account for the repeated-measures structure of the data.

**Results:**

Baseline treatment expectations did not significantly predict depressive symptoms at post-treatment. However, expectations measured at two weeks (T1) and four weeks (T2) significantly predicted depressive symptoms at T3. The results of the multiple regression analyses indicate that treatment expectations can predict changes in depressive symptoms, whereas the reverse relationship was not observed. The CLPM yielded results that were consistent with the regression analyses, supporting the robustness of the findings.

**Conclusions:**

Treatment expectations evolve throughout therapy and appear to function as an independent predictor of symptom improvement rather than merely reflecting symptom severity. Monitoring and addressing patient expectations early in treatment may enhance intervention outcomes. These findings support the inclusion of expectation-based strategies in IBIs to optimize engagement and effectiveness.

## Highlights

• Baseline treatment expectations did not predict post-treatment depressive symptoms
• Treatment expectations at two and four weeks were significant predictors, highlighting their role as *early process predictors* (EPPs).
• Prior treatment expectations influenced later depressive symptoms, not vice versa.
• Expectancy-enhancing strategies early in treatment could improve engagement and effectiveness in internet-based interventions.
• Monitoring and addressing patient treatment expectations dynamically throughout therapy may optimize outcomes

## 1 Introduction

As digital technologies continue transforming healthcare, internet-based interventions (IBI) have emerged as an effective and accessible solution for the treatment of many mental health conditions, including depression (Andersson and Berger, 2021; Hedman-Lagerlöf et al., 2023; Karyotaki et al., 2017; Moshe et al., 2021). Most evidence comes from studies investigating unguided or guided IBIs, in which the presentation of a self-help application, often based on cognitive-behavioral therapy (CBT), is combined with minimal but often weekly and text-based contact with a therapist. Existing literature indicates that guided interventions for depressive symptoms and depression result in lower dropout rates, improved adherence, and greater effectiveness compared to unguided interventions (Bur et al., 2022a, Bur et al., 2022b; Krieger et al., 2023; Karyotaki et al., 2021; Moshe et al., 2021), with benefits possibly especially pronounced in participants experiencing moderate to severe depressive symptoms (Karyotaki et al., 2017; Terhorst et al., 2024). However, even in the more effective guided interventions, over 40 % of participants do not respond in terms of a reliable change, leaving substantial room for optimizing IBIs for depression (Karyotaki et al., 2018).

One approach to improving therapeutic effectiveness is identifying variables predicting treatment outcomes. A predictor is a variable that can help anticipate outcomes in an intervention by directly influencing the outcome either across all treatment groups or within a specific group. It may later be identified as a moderator, mediator, or non-specific predictor in future research (Kraemer et al., 2002; Kraemer, 2008). Understanding predictors of outcome could improve effectiveness by enabling research-based tailoring of interventions to individual characteristics and needs (Barber, 2007; Donker et al., 2013).

A recent systematic review of predictors of treatment outcomes in IBI's for depression identified baseline depression severity as the strongest predictor, with higher baseline scores associated with better treatment outcomes (Sextl-Plötz et al., 2024). Beyond depression severity, patient treatment expectations have long been considered an important predictor of treatment outcome (Frank, 1961). Treatment expectations are beliefs and assumptions that patients hold about the outcomes of participating in therapy (Constantino et al., 2011). Previous studies have focused mainly on patient treatment expectations as *pre-treatment predictors (*PTP). These studies suggest that patients' initial beliefs about therapy outcomes can influence the therapeutic process and eventual success. For example, higher baseline treatment expectations have been linked to a better treatment alliance and improved clinical outcomes, particularly in conditions such as depression and anxiety (Vîslă et al., 2021). A meta-analysis of individual participant data from six randomized clinical trials (*n* = 491) comparing online and face-to-face psychological interventions for psychiatric and somatic conditions found that treatment expectations significantly predicted clinical outcomes (β = 0.27) at the end of therapy, with no moderating effect of treatment modality suggesting that expectancy effects are equally influential in both settings (Pontén et al., 2023). Regarding IBIs, Boettcher et al. (2013), for example, investigated patient treatment expectations in an internet-based self-help program for social anxiety and found that higher pre-treatment expectations predicted better adherence and outcomes. Similarly, Mira et al. (2019) explored the relationship between expectations and clinical outcomes in an internet-based intervention for depression and found that higher expectations were associated with greater improvements in depression symptoms and higher intervention completion rates. Zagorscak et al. (2020) also found that, in internet-based interventions for depression, treatment expectations were related to symptom change through task/goal ratings of the working alliance, suggesting that expectations can predict improvements in depression outcomes.

Despite the evidence supporting the role of treatment expectations in predicting therapy outcomes, findings remain mixed in the context of IBIs. While several studies have linked higher pre-treatment expectations to better adherence and symptom improvement, others, such as Thielecke et al. (2024), found no such effect. This inconsistency highlights the need to explore predictors beyond treatment expectations as PTP*.* One promising avenue is the examination of variables measured early in the treatment process, referred to as *early process predictors* (EPP; Hedman et al., 2016). Unlike PTP, EPPs can be used to inform therapeutic decisions on an ongoing basis and may be able to provide early information on which patients are responding to IBIs, and which may need to be offered an alternative form of treatment (Forsell et al., 2019). For example, meta-analyses have shown the predictive nature of early response to treatment (Beard and Delgadillo, 2019).

Patients' treatment expectations are likely to be shaped particularly during the initial therapy sessions, where they can be influenced by new information and remain dynamic rather than fixed constructs (Kirsch, 1985). Early sessions may be crucial for patients to develop an understanding of the therapy process, which in turn may change their expectations (Tsai et al., 2014; Vîslă et al., 2021). It can be assumed that if treatment expectations are measured before the treatment, the scores are more likely to reflect general assumptions about the treatment such as the treatment format, whereas if expectations are measured at a later stage, the scores are more likely to be influenced by actual experiences with the treatment, including its effects (Pontén et al., 2023). Given the dynamic nature of treatment expectations, exploring how these evolve from pretreatment over the first few sessions of therapy is important. This includes understanding the factors that predict changes in expectations and their relationship to symptom change. For example, patient characteristics such as gender, educational level, or the presence of previous depressive episodes have been associated with differences in expectations or their trajectories over time (Vîslă et al., 2019; Cohen et al., 2015; Grosse Holtforth et al., 2011). Furthermore, a study by Vîslă et al. (2021) showed that, on average, patients' expectations of treatment outcome increased linearly from pre-treatment to the end of treatment, with higher initial depression severity associated with lower expectations, an early increase in general self-efficacy associated with a flatter increase in expectations, and differences between therapists in the change in their patients' expectations. Treatment expectations may influence or be influenced by symptom changes during therapy.

Understanding EPPs, such as expectancies, in internet interventions for depression, could allow early personalized adjustments to treatment and therapy, increasing its effectiveness. To this end, this study conducts a secondary analysis of data from a factorial trial (Bur et al., 2022a, Bur et al., 2022b), focusing on the specific role of patient treatment expectations as pretreatment and EPP in an internet-based intervention for mild to moderate depression. Additionally, the study examines whether changes in depressive symptom levels were driven by changes in treatment expectations or whether changes in treatment expectations were driven by changes in depressive symptom levels over the course of treatment (baseline, at 2 weeks and at 4 weeks). By examining the temporal dynamics of treatment expectations as has been done in face-to face context by Meyerhoff and Rohan (2016), this study adds novel insights into how expectations develop and influence outcomes in internet-based therapy.

## 2 Methods

### 2.1 Study design

The secondary analysis utilized data from a randomized full factorial trial (Bur et al., 2022a, Bur et al., 2022b; Bur et al., 2021) investigating an internet-based problem-solving intervention. The design incorporated four experimental factors - diagnostic interview, motivational interviewing, guidance, and automated messages - each manipulated at two levels (present vs. absent), resulting in 16 experimental conditions (2 × 2 × 2 × 2). This present paper focuses on the variables of treatment expectancies (CEQ-8) and depressive symptoms (PHQ-9) at different time points. Furthermore, all analyses are conducted for the total sample and separately for the guided and unguided conditions, starting from the timepoint when guidance was introduced in the respective groups, as the factorial trial identified efficacy differences between them. The Canton of Bern Ethics Committee approved the study (2019–01795), and it was preregistered at ClinicalTrials.gov (NCT04318236).

### 2.2 Participants

A total of 317 participants with mild to moderate depressive symptoms were recruited from the community in Switzerland, Germany, and Austria through depression-related websites, radio interviews, self-help groups, Facebook groups, Google advertisements, and the website of the University of Bern (Switzerland). Inclusion criteria were 1) being 18 years of age, 2) indicating mild to moderate depressive symptoms on the Patient Health Questionnaire-9 (PHQ-9 score between 5 and 14), 3) written informed consent, 4) access to the internet and an email account, and 5) providing an emergency contact. Exclusion criteria were 1) reporting a present or past psychotic or bipolar disorder, or 2) indicating increased suicidal tendencies on the Suicidal Behavior Questionnaire-Revised (SBQ-*R* > 7). Participants receiving medication or psychotherapy were also eligible. The mean age of the participants was 38 years (SD = 13.66, range: 19–78). Most participants were female (71.8 %), single (62.3 %), and Swiss (51.0 %) or German (43.0 %). Most participants reported university education (58.9 %) and part-time or full-time employment (59.5 %). About a third of participants were receiving concurrent psychological treatment (29.8 %), and about a fifth were taking prescribed medication for mental disorders (20.3 %) at baseline.

### 2.3 Intervention

The internet-based intervention used in the trial was based on problem-solving therapy (Nezu et al., 2012). The intervention includes an introduction and 3 toolkits: (1) Feeling, (2) Thinking, and (3) Acting. Problem-solving therapy focuses on problems causing distress and problem-solving skills (Nezu et al., 2012). Participants were recommended to use the intervention for a minimum of 1 h per week. The total recommended program use was 8 weeks. For a more detailed description of the intervention, see Bur et al. (2021).

### 2.4 Procedure

After providing informed consent, participants were asked to complete a baseline assessment, and eligibility for the study was assessed. Following baseline assessment, participants were randomized to 1 of 4 groups (1, diagnostic interview and motivational interviewing module; 2, diagnostic interview; 3, motivational interviewing module; 4, no factor). Randomization was stratified for mild (PHQ-9: 5–9) or moderate (PHQ-9: 10–14) depressive symptoms. Participants then had to wait two weeks before starting with the intervention. During this time, and depending on the first randomization outcome, participants were asked to either wait, undergo a diagnostic interview, receive access to the preintervention motivational interviewing module, or receive both the interview and the preintervention motivational interviewing module. Participants were then randomized a second time to the following conditions: 1, guidance; 2, automated emails; 3, guidance and automated emails; 4, neither of these two factors. Further assessments were conducted after two weeks (T1), after four weeks (T2), after ten weeks (T3, post-assessment), and after 16 weeks (T4). The present study used the primary outcome at T3 and the expectations assessments at T0, T1, and T2.

### 2.5 Measures

A detailed description of all measures used can be found in the main results paper and the corresponding study protocol (Bur et al., 2021, Bur et al., 2022a, Bur et al., 2022b). The questionnaires for the study were administered over the internet.

#### 2.5.1 Primary outcome

The Patient Health Questionnaire-9 (PHQ-9) was the primary outcome measure. The PHQ-9 is a validated 9-item self-report tool for assessing depressive symptoms, with scores ranging from 0 to 27 (Kroenke et al., 2001). Post-intervention Cronbach's α ranged from 0.81 to 0.84 (Bur et al., 2022a, Bur et al., 2022b).

#### 2.5.2 Treatment expectancies

Treatment expectancy was assessed using the Credibility and Expectancy Questionnaire (CEQ-8; Devilly and Borkovec, 2000; German version: Walach et al., 2008). The CEQ-8 consists of two subscales: Items 1–3 assess the credibility of the treatment rationale, while items 4–6 measure outcome expectancies. Credibility reflects how logical and believable participants perceive the treatment to be, while expectancy captures both explicit and implicit beliefs about the likely success of the treatment. The total score, calculated by combining both subscales, is referred to in this study as the treatment expectancy or CEQ-8 score. The CEQ-8 was administered at baseline, after two weeks and four weeks. At baseline, the CEQ-8 subscales, Credibility and Expectancy, were highly correlated with each other (*r* = 0.501, *p* < .001) and with the total score (Credibility: *r* = 0.830, p < .001; Expectancy: *r* = 0.903, *p* < .001); hence, all analyses included the CEQ-8 total score as it has been done in previous research by Boettcher et al. (2013). Cronbach's α at baseline was 0.84.

#### 2.5.3 Statistical analysis

To examine the specific relationships between each timepoint of the CEQ-8 score and the dependent variable (PHQ-9 score post-treatment, T3), simple regression models were conducted while controlling for PHQ-9 at T0. These preliminary analyses provided an initial assessment of the magnitude and relevance of each predictor variable and helped to identify the specific time points at which the CEQ-8 score significantly predicted PHQ-9 outcome, controlling for baseline depressive symptoms.

To understand the interplay between treatment expectancy and depressive symptoms, a series of multiple regression analyses were conducted. These models were designed to examine whether treatment expectations predicted depressive symptoms or whether depressive symptoms influenced treatment expectations. A hierarchical entry method was used. In one set of models, PHQ-9 scores at different time points were used as the dependent variable, while in the other set, CEQ-8 scores at different time points were used as the dependent variable. To assess potential multicollinearity among the independent variables in the multiple regression model, the Variance Inflation Factor (VIF) and Tolerance was calculated for each predictor. A VIF value exceeding 5 or a Tolerance value below 0.2 was considered indicative of problematic multicollinearity (Eid et al., 2015). In addition to the multiple regression models, a cross-lagged panel model (CLPM) was conducted as a sensitivity analysis to validate the robustness of the regression results. The detailed model specification and results are provided in the Appendix. Analyses were conducted separately for the guidance and non-guidance conditions, as there were significant differences between guidance vs. no guidance in the main study (Bur et al., 2022a, Bur et al., 2022b). Since participants were randomized into guidance vs. no guidance after T1, all analyses before T2 were performed solely for the total sample. Analyses stratified by guidance and no-guidance conditions are presented in the Appendix. Unless otherwise stated, all analyses in the main text refer to the total sample.

All statistical analyses were conducted using Jamovi Version 2.3 (The Jamovi Project, 2024). In addition, the CLPM was estimated in R (R Core Team, 2025) using the lavaan package (Rosseel, 2012).

## 3 Results

### 3.1 Descriptive analysis

Descriptive information on means and standard deviations of depressive symptoms and CEQ-8 score, over time are reported in Table 1. Descriptive results concerning patient characteristics have been reported in previous studies (Bur et al., 2022a, Bur et al., 2022b). There were no pre-treatment differences between the guided and the unguided groups regarding demographics, depressive symptoms, current psychotherapeutic treatment, and current medication.

**Table 1:** Observed means and standard deviation of depressive symptoms and treatment expectancy.

Measures	N	Mean	SD
Depression symptoms			
At baseline (T0)	316	9.30	2.56
At 2 weeks (T1)	302	9.20	3.84
At 4 weeks (T2)	258	8.15	3.80
At 10 weeks (T3)	209	7.20	4.51

Treatment expectancy			
At baseline (T0)	316	32.70	8.17
At 2 weeks (T1)	302	32.95	8.41
At 4 weeks (T2)	254	31.73	10.38

Note. T0= Baseline; T1 = 2 weeks after baseline; T2 = 4 weeks after baseline; T3 = 10 weeks after baseline (post-treatment)*.*

### 3.2 Intervention outcomes

The results of the factorial trial were previously reported by Bur et al., 2022a, Bur et al., 2022b. Both guided (*d* = 0.72) and unguided participants (*d* = 0.38) experienced a statistically significant reduction in depressive symptoms at post-treatment with a small but statistically significant between-group effect, favoring guided interventions (*d* = 0.15). No significant differences were found between the other experimental groups.

### 3.3 Identifying predictors

To explore the specific relationships between each predictor and the dependent variable, simple regressions were calculated to identify the predictors explaining most of the total variance. Results are reported in Table 2. VIF values for all predictors ranged from 1.00 to 1.07, suggesting minimal multicollinearity and confirming the independence of predictors in the regression model.

**Table 2:** Simple regressions with depressive symptoms at T3 as dependent variable.

Variable	Measurement time	B (SE)	*p*	R^2^	R^2^ adj.
PHQ-9	T0	0.70 (0.11)	<0.001	0.153	0.149
CEQ-8^a^	T0	−0.07 (0.04)	0.054	0.168	0.160
	T1	−0.09 (0.03)	0.013	0.178	0.170
	T2	−0.13 (0.03)	<0.001	0.235	0.228

a Simple regressions controlled for PHQ-9 at baseline. PHQ-9, Patient Health Questionnaire; CEQ-8, Credibility and Expectancy Questionnaire; T0 = Baseline; T1 = 2 weeks after baseline; T2 = 4 weeks after baseline; T3 = 10 weeks after baseline (post-treatment).

#### 3.3.1 Depressive symptoms

Depressive symptoms at baseline significantly predicted depressive symptoms at post-treatment (T3), with higher baseline PHQ-9 scores being associated with higher PHQ-9 scores at post-treatment (Table 2). Depressive symptoms at baseline explained 14.9 % of the variance in depressive symptoms at T3.

#### 3.3.2 Treatment expectancy

CEQ-8 score at baseline (T0) did not significantly predict depressive symptoms at T3. At two weeks (T1), CEQ-8 score was a significant predictor of depressive symptoms at T3, as shown in Table 2. CEQ-8 score at four weeks (T2) demonstrated predictive significance across the total sample (Table 2) and the guided and unguided sample (Appendix A). Higher CEQ-8 scores, reflecting stronger credibility and/or expectancy of the intervention, were associated with lower PHQ-9 scores, indicating an improvement in depressive symptoms. CEQ-8 score at four weeks (T2) accounted for 22.8 % of the variance in the total sample, 26.2 % in the guided sample, and 21.4 % in the unguided sample (Appendix A).

### 3.4 Understanding the interplay between treatment expectancy and depressive symptoms

To understand the interplay between treatment expectancy and depressive symptoms, a series of multiple regression analyses were conducted. These models were designed to examine whether treatment expectations predicted depressive symptoms or whether depressive symptoms influenced treatment expectations. To explore these relationships, the analyses assessed whether earlier treatment expectations predicted later symptom levels and whether prior symptoms influenced subsequent treatment expectations while controlling for preceding levels of each variable. The analyses included five models, with three focusing on PHQ-9 as the dependent variable and two focusing on CEQ-8 score as the dependent variable.

#### 3.4.1 Depressive symptoms as dependent variable at two, four and ten weeks

CEQ-8 score at T0 did not significantly predict depressive symptoms at T1. However, CEQ-8 score at T1 (*β*_T1_ = −0.10) and T2 (*β*_T2_ = −0.12) significantly predicted PHQ-9 scores at the subsequent time point (i.e., T2 and T3). PHQ-9 score significantly predicted depressive symptoms across all time points (*β*_T0_ = 0.52; *β*_T1_ = 0.73; *β*_T2_ = 0.61; Table 3).

**Table 3:** Multivariate regressions with PHQ-9 scores and CEQ-8 scores at different time points as predictors and dependent variables.

DV	Predictors	*B*	*SE*	β	*p*	Change in R^2^	R^2^	Adj. R^2^
PHQ at T1	Intercept	2.50	1.05		0.018		0.273	0.268
	PHQ at T0	0.78	0.07	0.52	<0.001			
	CEQ at T0	−0.02	0.02	−0.04	0.467	0.001		
PHQ at T2	Intercept	3.10	0.77		<0.001		0.540	0.536
	PHQ at T1	0.72	0.04	0.73	<0.001			
	CEQ at T1	−0.05	0.02	−0.10	0.018	0.010*		
PHQ at T3	Intercept	2.92	1.08		0.007		0.420	0.414
	PHQ at T2	0.70	0.06	0.61	<0.001			
	CEQ at T2	−0.05	0.02	−0.12	0.037	0.013*		
CEQ at T1	Intercept	6.42	1.75		<0.001		0.576	0.574
	PHQ at T0	0.07	0.12	0.02	0.587			
	CEQ at T0	0.79	0.04	0.76	<0.001	0.567***		
CEQ at T2	Intercept	7.08	2.38		0.003		0.406	0.402
	PHQ at T1	−0.17	0.13	−0.06	0.197			
	CEQ at T1	0.79	0.06	0.64	<0.001	0.404***		

Note. DV = dependent variable; PHQ = PHQ-9 score; CEQ = CEQ-8 total score; T0 = Baseline; T1 = 2 weeks after baseline; T2 = 4 weeks after baseline; T3 = 10 weeks after baseline (post-treatment); * p < .05, ** p < .01, *** p < .001. All models were calculated for the total sample.

#### 3.4.2 Treatment expectancy as dependent variable at two and four weeks

PHQ-9 score did not significantly predict treatment expectancies at any time point (Table 3). Treatment expectancy emerged as a significant predictor of treatment expectancies across all time points (*β*_T0_ = 0.76; *β*_T1_ = 0.64).

Appendix B, Appendix C describe the control for potential differences in the *guidance* condition. At the relevant measurement time points (from T1), the results for the guidance and non-guidance conditions did not differ. Findings from the cross-lagged panel model (CLPM) were consistent with the regression analyses (see Appendix D).

## 4 Discussion

In this study, a secondary analysis of data from a factorial trial (Bur et al., 2022a, Bur et al., 2022b), the role of treatment expectations in an internet-based treatment for depressive symptoms was examined. First, the role of treatment expectations as *pre-treatment predictor* and *early process predictor* was analyzed using simple regression analyses. Second, the temporal interplay between treatment expectations and depressive symptom levels was investigated by investigating whether earlier expectations predicted subsequent symptom levels and whether earlier symptoms influence later expectations.

The simple regression analyses showed that treatment expectations at baseline did not significantly predict depressive symptoms at ten weeks. This finding aligns with previous research in face-to-face settings suggesting that baseline expectations may not fully capture patients' evolving beliefs as they engage with the intervention (Tsai et al., 2014; Vîslă et al., 2021). In contrast, treatment expectations assessed at two weeks was significantly associated to depressive symptoms. Notably, treatment expectations at four weeks consistently showed significant association across all samples. Whether participants received guidance or not does not appear to have an impact on whether treatment expectancy is a significant predictor of depressive symptoms at four weeks. A possible explanation for the lack of a significant association between baseline treatment expectations and posttreatment depressive symptoms is that expectations assessed prior to treatment may reflect general attitudes toward therapy rather than specific, experience-based beliefs about the intervention (Meyerhoff and Rohan, 2016; Vîslă et al., 2021). Such pre-treatment expectations are often shaped by hope or assumptions and may lack grounding in the actual treatment experience. In contrast, expectations assessed during treatment are more likely to be informed by direct engagement with the intervention and thus better reflect participants' perceptions of its relevance and effectiveness. This shift from generalized to experience-based expectations may explain why expectations at later time points are more predictive of outcomes (Pontén et al., 2023).These results highlight the importance of viewing treatment expectations not as a static baseline measure but as a dynamic construct that evolves throughout therapy (Kirsch, 1985). This dynamic nature of treatment expectations highlights the need to monitor and address expectations throughout the treatment process and potentially also the chance for expectancy-fostering interventions early in treatment to augment patient treatment expectations and thus depressive symptoms. Previous research has shown that acceptance-facilitating interventions can augment patient acceptance (Ebert et al., 2015).

Furthermore, the results suggest that allowing participants to experience the intervention may be beneficial before their expectations gain predictive value. This could be because participants' initial expectations before the actual treatment started are often unrealistic (Constantino et al., 2011). As their understanding of the intervention becomes more concrete, their expectations may adjust to be more realistic, thereby increasing their predictive value. Consequently, expectations only attain predictive relevance after a certain period, as observed in the present study from two weeks onward.

The predictive value of treatment expectancy after two and four weeks suggests that monitoring treatment expectations during these early stages can offer critical insights into patient trajectories. Therapists could proactively address and foster positive expectations as early as two weeks into the program. From a broader perspective, these findings advocate for integrating regular assessments of treatment expectations into internet-based interventions. Identifying patients with low CEQ-8 scores early in the treatment process could enable timely adjustments, such as personalized feedback or supplemental support, to enhance adherence and outcomes.

The results of the multiple regression analyses indicate that treatment expectations can predict changes in depressive symptoms, whereas the reverse relationship was not observed. These findings may help to answer the question of whether treatment expectations merely reflect symptom severity or represent an independent construct influencing treatment outcomes. The present findings suggest that treatment expectations at two and four weeks predict later depressive symptoms rather than simply resulting from them. This perspective supports the idea that treatment expectations may function as an independent process factor rather than merely reflecting changes in depressive symptoms.

These findings represent an initial step toward systematically examining the causal relationship between treatment expectations and depressive symptom improvement in IBIs. The precise mechanisms underlying this interaction remain unclear. As demonstrated in face-to-face therapy (Meyer et al., 2002), mediating factors such as the therapeutic alliance should be explored in the context of IBIs to better understand these dynamics. The present analyses do not establish causality, as unmeasured third variables, such as therapeutic alliance, may influence both subsequent treatment expectations and symptom changes. Rather, they offer a preliminary perspective on this interplay and suggest a possible direction for clinical practice. Future research should incorporate additional theoretically relevant variables, assessed alongside treatment expectations and symptoms, to further elucidate these mechanisms.

Building on our findings, integrating routine assessments of treatment expectations into internet-based interventions could enable early personalization of treatment trajectories. For instance, participants who report low expectations at two or four weeks might benefit from targeted strategies aimed at enhancing their engagement and belief in the treatment, such as motivational messages, additional psychoeducation, or early therapist contact, even in otherwise unguided programs.

Moreover, identifying individuals with persistently low expectations could prompt a shift toward more intensive formats or a different therapeutic approach altogether. Personalized adaptation might also involve adjusting the presentation of treatment rationale or tailoring content more closely to individual needs to boost perceived credibility.

In practice, platforms could be designed to automatically flag low CEQ-8 scores, triggering predefined support modules or alerts for therapist review. Embedding such adaptive mechanisms could increase treatment engagement and enhance outcomes, particularly for those at risk of dropout or nonresponse.

Our findings therefore support the inclusion of treatment expectations not only as an outcome predictor, but as a clinically actionable process variable that can guide timely adjustments to maximize intervention effectiveness.

### 4.1 Limitations

This study has notable strengths such as the large sample size and the collection of variables like treatment expectations at multiple time points. However, certain limitations must be acknowledged. First, the sample was self-selected from the community, limiting the findings' generalizability to clinical settings. Second, the study is further restricted to participants with mild to moderate depressive symptoms, leaving uncertainty about whether these patterns extend to more severe clinical populations or alternative treatment modalities. Third, the sample was more educated than the general population, which may influence the applicability of the results to broader demographic groups. Fourth, relying on self-reported measures introduces the potential for response biases. Incorporating clinician-administered assessments would have strengthened the study's validity. Furthermore, multiple statistical tests were conducted without formal correction for multiple comparisons, as our analyses were hypothesis-driven; however, this increases the risk of Type I error and the results should therefore be interpreted with this in mind. Lastly, exploring moderators such as demographic variables, program usage or therapeutic alliance, could provide deeper insights into the drivers of successful outcomes in internet-based interventions.

## 5 Conclusion

In conclusion, this study illustrates the importance of assessing and nurturing treatment expectations during the early stages of internet-based interventions. While baseline treatment expectations offer limited predictive value, treatment expectancy at two and four weeks stands out as an *early process predictor*. Furthermore, the findings indicate that treatment expectations predict changes in depressive symptoms rather than simply reflecting them, reinforcing the idea that expectations serve as an independent process factor. These findings emphasize the potential of utilizing evolving treatment expectations to enhance therapeutic outcomes, promoting a more dynamic and continuous assessment approach. Instead of considering treatment expectations as static baseline characteristics, tracking their progression over time and exploring their interaction with symptom changes could further personalize internet-based interventions, ultimately improving treatment engagement and outcomes.

## Abbreviations

PHQ: Patient Health Questionnaire
CEQ: Credibility and Expectancy Questionnaire

## Declaration of Generative AI and AI-assisted technologies in the writing process

During the preparation of this work the author(s) used Chat GPT and Grammarly in order to improve the language (grammar, spelling and punctuation) of the manuscript. After using these tools, the author(s) reviewed and edited the content as needed and take full responsibility for the content of the published article.

## Funding sources

Funding for the project was provided by the Department of Clinical Psychology and Psychotherapy at the 10.13039/100009068University of Bern, Bern, Switzerland.

## Declaration of competing interest

The authors declare the following financial interests/personal relationships which may be considered as potential competing interests: Jan Philipp Klein reports a relationship with 10.13039/501100003107German Federal Ministry of Health that includes: funding grants. Jan Philipp Klein reports a relationship with GAIA Oberberg that includes: speaking and lecture fees. Jan Philipp Klein reports a relationship with Boehringer, Ethypharm, GAIA, sympatient that includes: consulting or advisory. Jan Philipp Klein reports a relationship with Beltz, Elsevier, Hogrefe and Springer that includes: speaking and lecture fees. Jan Philipp Klein (co-author) is past president of the CBASP network (now DsG-CBASP) and serves as vice chairman of the chapter “Digital Psychiatry” of the German Psychiatric Association (DGPPN).

## Figures and Tables

**Appendix A:** Simple regression guidance vs non-guidance comparison.

Sample	Variable	Measurement time	B (SE)	*p*	R^2^	R^2^ adj.
Guided	PHQ	T0	0.656 (0.129)	<0.001	0.193	0.186
	CEQ	T2	−0.102 (0.030)	0.001	0.275	0.262
Unguided	PHQ	T0	0.770 (0.196)	<0.001	0.139	0.130
	CEQ	T2	−0.165 (0.046)	<0.001	0.231	0.214

Note. Simple regressions with PHQ T3 as dependent variable, controlled for PHQ-9 at baseline. PHQ-9, Patient Health Questionnaire; CEQ-8, Credibility and Expectancy Questionnaire; PHQ = PHQ-9 score; CEQ = CEQ-8 total score; T0 = Baseline; T1 = 2 weeks after baseline; T2 = 4 weeks after baseline; T3 = 10 weeks after baseline (post-treatment).

**Appendix B:** Multiple regressions analysis in the guided sample.

DV	Predictors	B	SE	β	*p*	Change in R^2^	R^2^	Adj. R^2^
PHQ at T1	Intercept	4.282	1.550		0.006		0.168	0.157
	PHQ at T0	0.585	0.108	0.409	<0.001			
	CEQ at T0	−0.013	0.034	−0.029	0.700	0.001		
PHQ at T2	Intercept	3.899	1.028		<0.001		0.421	0.421
	PHQ at T1	0.567	0.060	0.644	<0.001			
	CEQ at T1	−0.031	0.025	−0.084	0.281	0.007		
PHQ at T3	Intercept	1.588	1.160		0.174		0.494	0.485
	PHQ at T2	0.792	0.082	0.675	<0.001			
	CEQ at T2	−0.044	0.025	−0.120	0.088	0.014		
CEQ at T1	Intercept	5.897	2.507		0.020		0.605	0.599
	PHQ at T0	−0.029	0.174	−0.009	0.868			
	CEQ at T0	0.831	0.055	0.778	<0.001	0.605***		
CEQ at T2	Intercept	4.657	3.166		0.144		0.466	0.457
	PHQ at T1	0.066	0.188	0.023	0.727			
	CEQ at T1	0.804	0.078	0.681	<0.001	0,464***		

Note. DV = dependent variable; PHQ = PHQ-9 score; CEQ = CEQ-8 total score; T0 = Baseline; T1 = 2 weeks after baseline; T2 = 4 weeks after baseline; T3 = 10 weeks after baseline (post-treatment); * p < .05, ** p < .01, *** p < .001.

**Appendix C:** Multiple regression analysis in the unguided sample.

DV	Predictors	B	SE	β	*p*	Change in R^2^	R^2^	Adj. R^2^
PHQ at T1	Intercept	0.625	1.407		0.658		0.396	0.388
	PHQ at T0	0.985	0.100	0.627	<0.001			
	CEQ at T0	−0.018	0.031	−0.038	0.556	0.001		
PHQ at T2	Intercept	2.585	1.108		0.021		0.653	0.648
	PHQ at T1	0.870	0.058	0.800	<0.001			
	CEQ at T1	−0.066	0.029	−0.118	0.027	0.014*		
PHQ at T3	Intercept	4.472	1.943		0.024		0.380	0.367
	PHQ at T2	0.632	0.098	0.559	<0.001			
	CEQ at T2	−0.066	0.044	−0.130	0.138	0.015		
CEQ at T1	Intercept	6.996	2.488		0.006		0.546	0.540
	PHQ at T0	0.160	0.177	0.050	0.368			
	CEQ at T0	0.741	0.055	0.739	<0.001	0.545***		
CEQ at T2	Intercept	9.600	3.646		0.010		0.356	0.345
	PHQ at T1	−0.388	0.188	−0.150	0.041			
	CEQ at T1	0.765	0.096	0.578	<0.001	0.334***		

Note. DV = dependent variable; PHQ = PHQ-9 score; CEQ = CEQ-8 total score; T0 = Baseline; T1 = 2 weeks after baseline; T2 = 4 weeks after baseline; T3 = 10 weeks after baseline (post-treatment); * p < .05, ** p < .01, *** p < .001.

**Appendix D:**
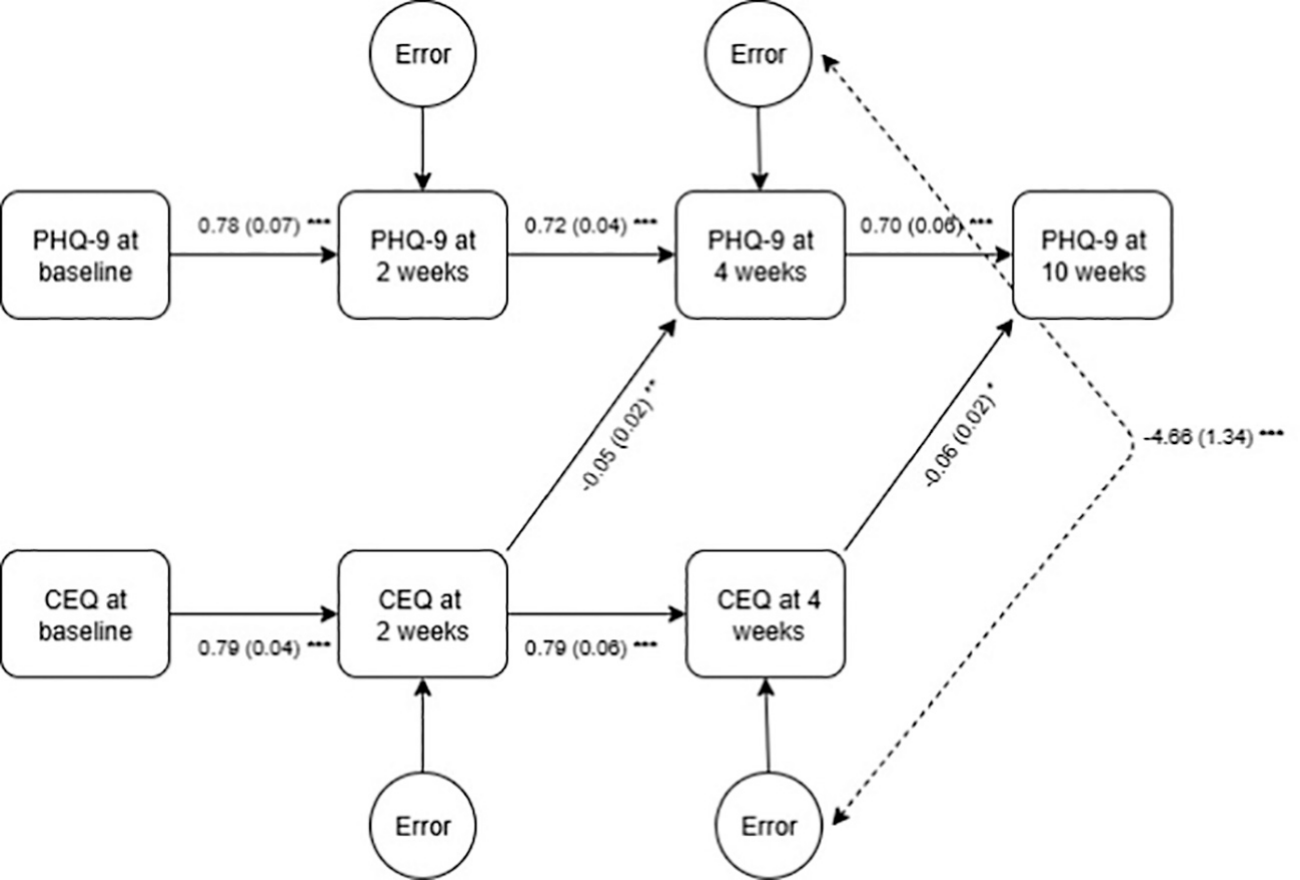
Cross-lagged panel model (CLPM) of treatment expectations (CEQ) and depressive symptoms (PHQ-9) across baseline, 2, 4, and 10 weeks in the total sample. Note. *1* *** = *p* < .0001,** = *p* < .01, * = *p* < .05. Only significant paths are displayed (standardized coefficients with standard errors in parentheses). Autoregressive and cross-lagged effects were estimated; results were fully consistent with the multiple regression analyses. This model was conducted as a sensitivity analysis and demonstrated an acceptable to good model fit, χ^2^(8) = 25.24, *p* = .001, CFI = 0.98, TLI = 0.95, RMSEA = 0.08 (90 % CI [0.05, 0.12]), SRMR = 0.04.

## Data Availability

The data sets generated and analyzed during the study are available from the corresponding author on reasonable request.
